# Supplementary material for: Glioblastoma Embryonic-like Stem Cells Exhibit Immune-Evasive Phenotype
Source: Cancers (Basel). 2022 Apr 21;14(9):2070. doi: 10.3390/cancers14092070 (PMC9104850; doi:10.3390/cancers14092070)
Supplement: Supplementary file 1 [file cancers-14-02070-s001.zip › cancers-1675417-supplementary.pdf]

Supplementary Materials: Glioblastoma Embryonic-like Stem Cells Exhibit Immune-Evasive Phenotype

Borja Sesé, Sandra Íñiguez-Muñoz, Miquel Ensenyat-Mendez, Pere Llinàs-Arias, Guillem Ramis, Javier I. J. Orozco, Silvia Fernández de Mattos, Priam Villalonga and Diego M. Marzese

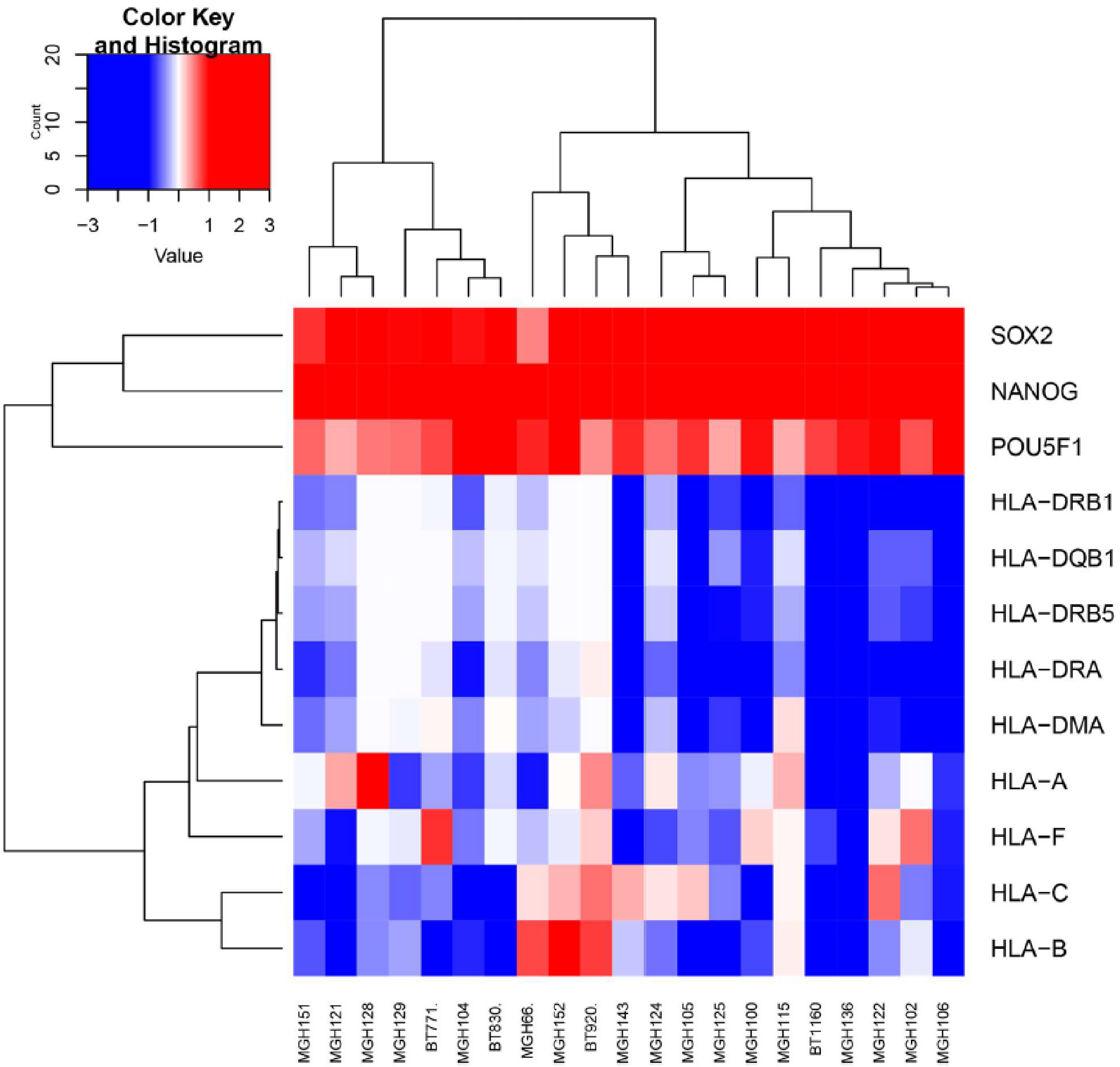

Figure S1. Heatmap displaying OSN and HLA gene expression fold changes between c-GSCs and the remaining cells from GBM tumor bulk in each patient. Genes with mean expression below 0.1 were removed from the analysis.

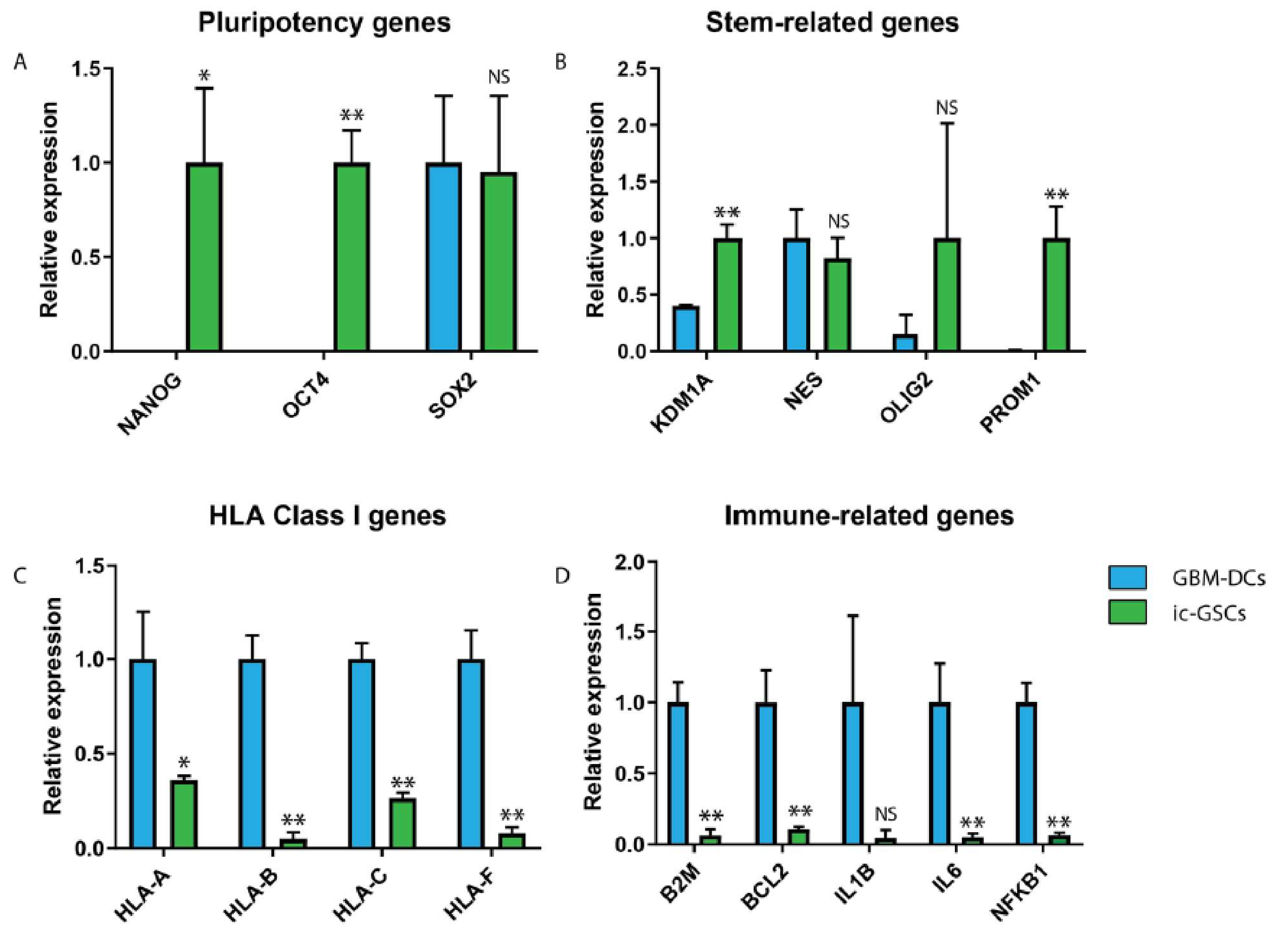

Figure S2. Bar plot showing relative expression levels of (**A**) pluripotency, (**B**) stem-related,(**C**) HLA Class I, (**D**) HLA Class I. (*p < 0.05; **p < 0.01; NS = non-significant differences).

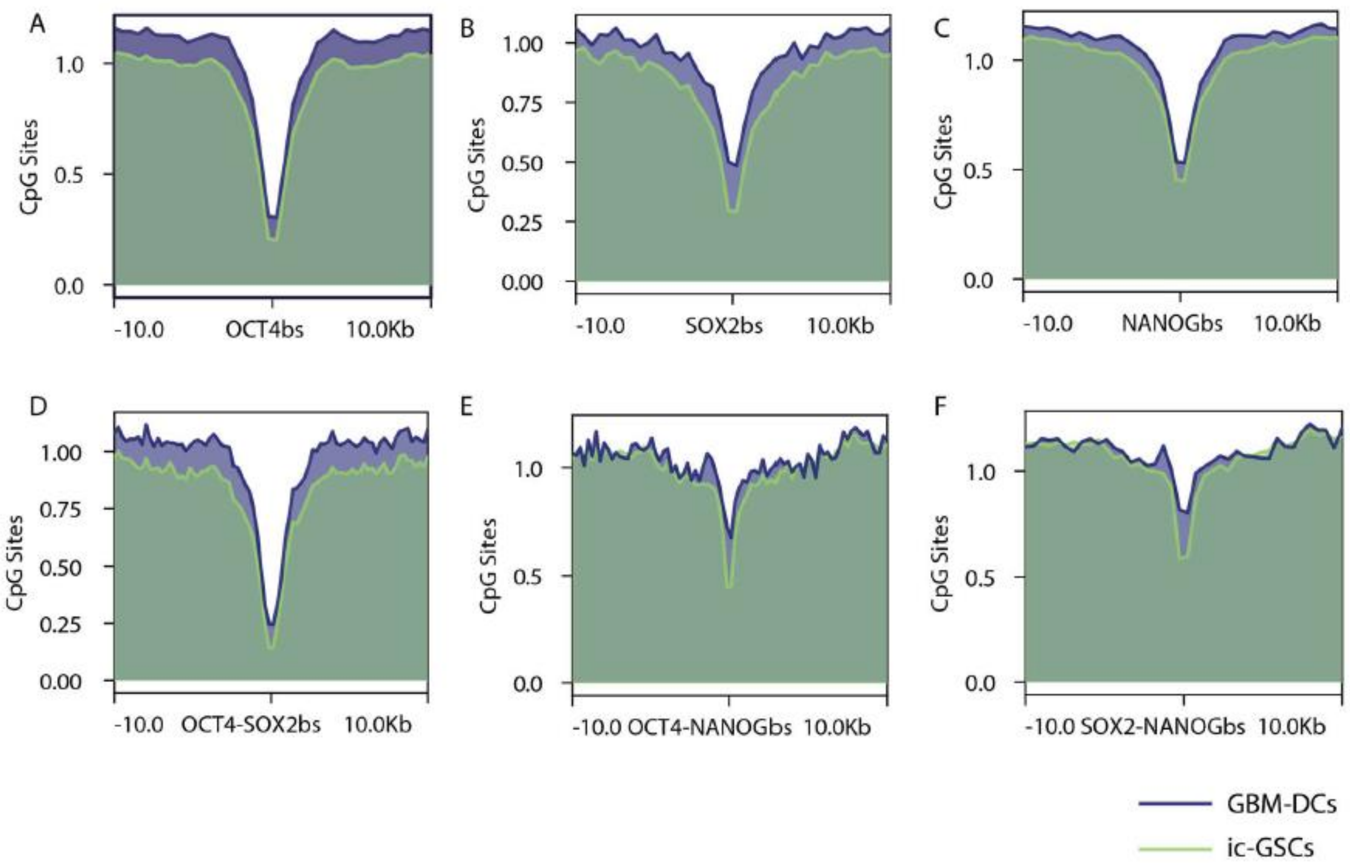

Figure S3. Profile plot representing the DNAm levels of GBM-DCs (blue) and ic-GSCs (green) around (±10kb) consensus (**A**) OCT4, (**B**) SOX2, (**C**) NANOG, (**D**) OCT4-SOX2, (**E**) OCT4-NANOG, and (**F**) SOX2-NANOG.

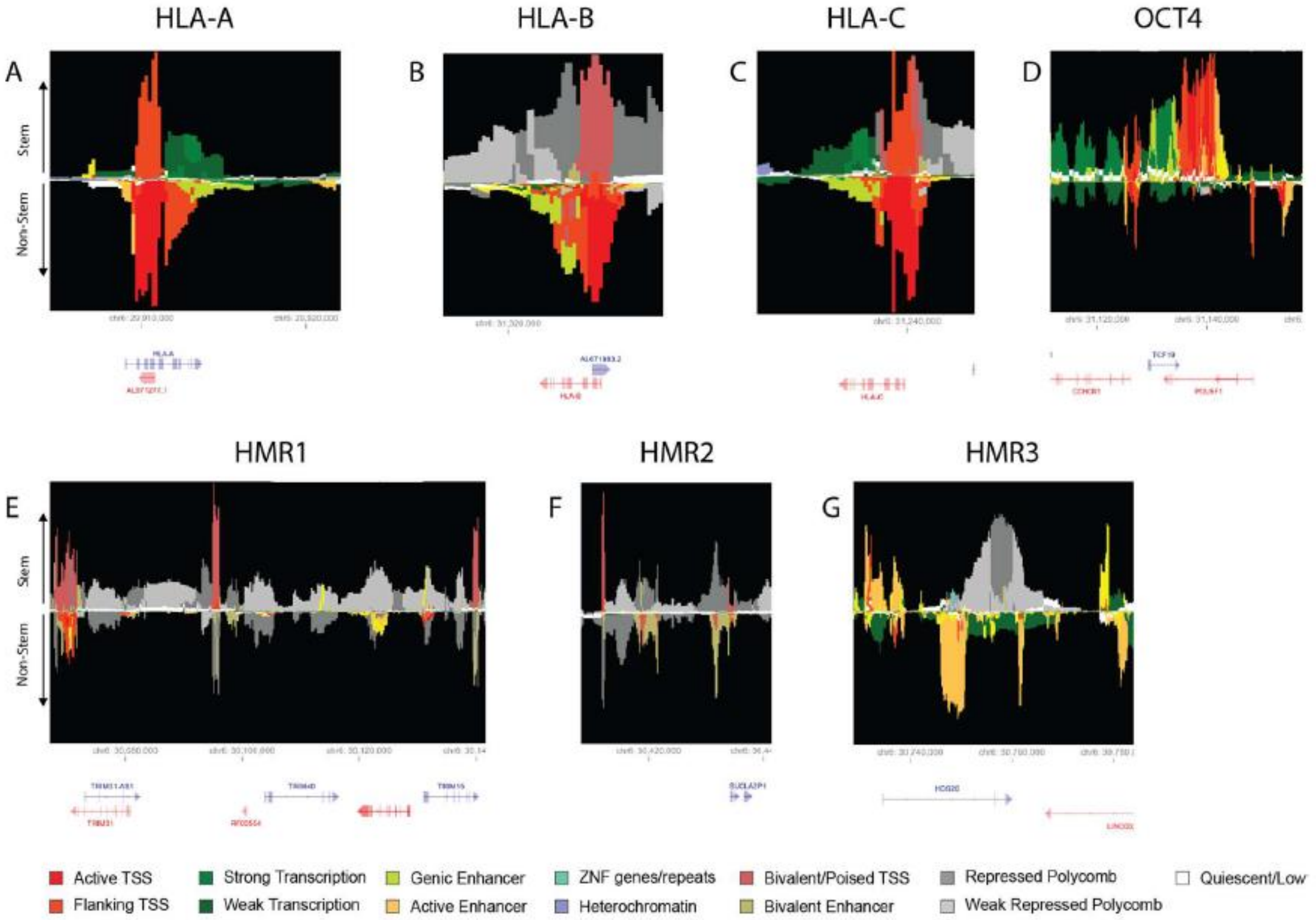

Figure S4. Chromatin state in stem versus non-stem biospecimens at (**A**) HLA-A, (**B**) HLA-B, (**C**) HLA-C, (**D**) OCT4 (POU5F1) genes, and (**E**–**G**) hypermethylated regions (HMR) in ic-GSCs.
